# Anaphase II processes contribute to aneuploidy in aging oocytes

**DOI:** 10.18632/aging.204640

**Published:** 2023-03-30

**Authors:** Anna Kouznetsova

**Affiliations:** 1 Department of Cell and Molecular Biology, Karolinska Institutet, Stockholm 17177, Sweden

**Keywords:** age-dependent aneuploidy, meiosis, oocyte, second meiotic division, segregation

Aneuploidy, or an incorrect number of chromosomes, is consistently associated with cell malfunction. Aneuploid germ cells develop into embryos with incorrect number of chromosomes, resulting in miscarriage or birth of an individual with developmental disorders, e.g., Down syndrome. Unfortunately, human female germ cells – oocytes – are particularly prone to aneuploidy. Aging aggravates the defect and provokes a drastic increase in aneuploidy level from 20–25% at 20 years to 50–60% in 40–50 years old women [1], which imposes a significant burden on individuals and the society.

Aneuploidy results from segregation errors during cell division. Oocytes undergo a meiotic type of division, comprised of two rounds of chromosome segregation events, resulting in formation of haploid eggs. Studies have identified multiple factors that contribute to age-dependent aneuploidy during the first meiotic division (MI) [2]. We know, however, much less about the chromosome segregation process during the second meiotic division (MII), even though approximately 40% of identified age-dependent segregation errors in humans are introduced during the second meiotic division following an error-free first meiotic division [3].

Aging also affects the accuracy of the chromosome segregation process in germ cells in female mice. We have analysed the chromosome segregation processes in oocytes derived from young adult (11–13 weeks old) and aged (47–50 weeks old) mice during the second meiotic division. The aged oocytes did not display an increase in aneuploidy on MI completion, but the level of aneuploidy was significantly elevated in haploid eggs following MII completion, from 4 ± 4.1% observed at 11–13 weeks, to 29 ± 7.6% at 47–50 weeks [4].

**Figure 1:**
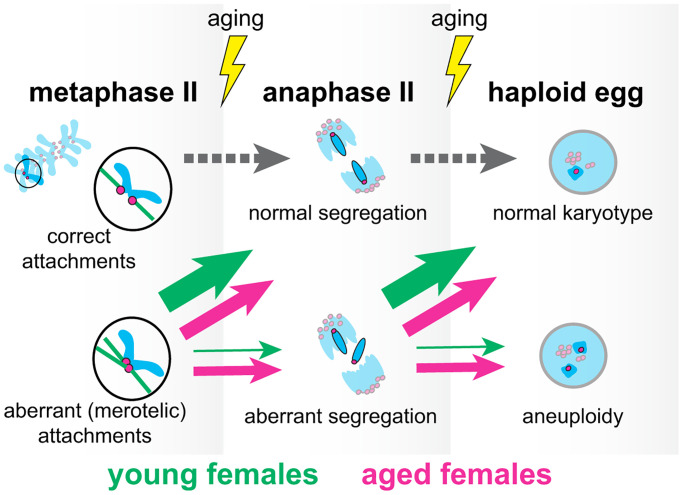
Aneuploidy in mammalian eggs increases with age due to an impaired ability to resolve aberrant kinetochore-microtubule attachments post metaphase II stage.

Aging did not affect chromosome behaviour at the metaphase II stage: the positions of chromosomes relative to the spindle equator and the spindle axis and the speed and orientation of chromosomes were similar to what previously been documented for chromosomes in young oocytes [5]. Furthermore, at the metaphase II stage the centromere/kinetochore architecture, the kinetochore-microtubule attachments and the structure and stability of the spindle were the same in oocytes from young and aged mice. In contrast, aged oocytes exhibited a strongly increased level of chromosome segregation abnormalities at anaphase II: oocytes collected from aged mice showed more aberrant anaphase II segregation patterns (an increase from 15% in young mice to 55% in aged mice) and an increased percentage of abnormally segregating chromosomes that contributed to formation of aneuploid gametes (an increase from 10% in young mice to 30% in aged mice). Consequently, the number of chromosomes in aged oocytes that contribute to aneuploidy is increased by an order of magnitude, from 0.02–0.04 per oocyte in young females to 0.2–0.4 per oocyte in aged females (Figure 1).

The distances between sister kinetochores at the metaphase II stage for aberrantly segregating chromosomes were reduced from 1.3 ± 0.7 μm in young mice to 0.8 ± 0.3 μm in aged mice [4]. Considering that the distribution of all inter-kinetochore distances at the metaphase II stage is similar in young and aged oocytes, the observed reduction indicates that aging primarily affects segregation of chromosomes with small distances between sister kinetochores. The reduced inter-kinetochore distances are associated with so called merotelic kinetochore-microtubule attachments, when a sister kinetochore is attached to the microtubules emanating from the opposite spindle poles. Chromosomes with merotelic attachments will persist at the spindle midzone at anaphase II until one of the two MTs dissolves, allowing the lagging sister chromatid to segregate to one of the two spindle poles [6]. We hypothesise that the age-dependent defect observed here results from a failure to correctly resolve aberrant kinetochore-microtubule attachments during the anaphase II stage. The importance of an anaphase mechanism for correctly segregating lagging chromatids has been demonstrated in several models [7, 8], but the molecular details of the process in oocytes remain to be elucidated.

In summary, this study reveals a novel factor contributing to age-dependent aneuploidy in mammalian oocytes, namely, a defect in correcting kinetochore-microtubule attachments at a post metaphase II stage. This defect is among the earliest examples of age-affected processes, surfacing before any signs of aging become visible at the metaphase II stage. The observed age-dependent defects are due to yet uncharacterized factors located at the spindle midzone that promote resolution of merotelic attachments in oocytes in young females but fail to execute these functions in oocytes in aged females. Our results reveal that post-metaphase II processes have critical impact on age-dependent aneuploidy in mammalian eggs and indicate new potential intervention points for reducing age-dependent aneuploidy in mammalian eggs.
